# HES5 Activates Long Noncoding RNA UCA1 to Induce Colorectal Cancer Progression by Modulating miR-185/NOTCH3 Signaling

**DOI:** 10.1155/2021/7249818

**Published:** 2021-10-25

**Authors:** Mingming Wang, Qinlin Zheng, Zhengfei Zhao, Hong Deng, Qiang Zhang, Hui Yao

**Affiliations:** ^1^Department of General Surgery (Gastrointestinal Surgery), The Affiliated Hospital of Southwest Medical University, China; ^2^Department of Obstetrics and Gynecology, The Affiliated Hospital of Southwest Medical University, China

## Abstract

Colorectal cancer (CRC) is one of the most common diagnosed cancers around the world. The poor prognosis and high fatality caused by metastasis are still the challenges for clinical treatment. Therefore, it is promising to clarify the detailed molecular mechanism of CRC metastasis. Accumulating evidences indicate that long noncoding RNAs (lncRNAs) play important roles in cancer progression including CRC. In this study, the function of lncRNA UCA1 was investigated. UCA1 was confirmed to be highly expressed in colorectal cancer. Moreover, the UCA1 expression level was positively related to tumor stages. Silencing UCA1 showed inhibitory effect on cell proliferation and metastasis. Both UCA1 and NOTCH3 were validated as direct targets of miR-185. Silencing UCA1 repressed NOTCH3 expression through the miR-185 sponge. NOTCH3 was found to be highly expressed in CRC patients and positively related to UCA1 expression. Furthermore, HES5 was verified as a transcription factor of UCA1, which induced UCA1 expression. In conclusion, UCA1 is a direct target of HES5. UCA1 promotes CRC metastasis through regulating the miR-185/NOTCH3 axis.

## 1. Introduction

Colorectal cancer (CRC) is one of the most commonly diagnosed cancers around the world [1]. In year 2018, nearly 1.8 million new CRC cases were diagnosed and over 800,000 deaths occurred due to metastasis [2]. Although the treatments for CRC have been improved recently, the poor prognosis and high fatality are still the challenges because of metastasis. Therefore, it is promising to clarify the detailed molecular mechanisms of CRC metastasis and provide potential therapeutic targets. Accumulating evidences indicate that long noncoding RNAs (lncRNAs) play important roles in cancer progression including CRC [3]. Thus, our study will investigate the function of lncRNA in CRC metastasis.

lncRNA is a type of noncoding RNA with more than 200 nucleotides lacking protein coding ability [4]. lncRNA exhibits multiple functions including chromatin modification, gene transcription, and posttranscription regulation [5]. The lncRNA urothelial cancer-associated 1 (UCA1) was first identified from bladder cancer cell line BLZ-211. It locates at chromosome 19p13.12 with around 2280 nucleotides [6]. UCA1 functions as a tumorigenic lncRNA in different cancers such as bladder cancer [7], breast cancer [8], lung cancer [9], melanoma [10], and colorectal cancer [11]. Furthermore, UCA1 is considered as a biomarker indicating poor prognosis of patients, in which metastasis and drug resistance occur [12]. Previous studies have shown that UCA1 promotes CRC metastasis through different molecular mechanisms including the lncRNA-miRNA-mRNA regulatory axis. Nevertheless, it is still imperfect of the whole landscape of UCA1 mechanisms involving different miRNAs in CRC metastasis. Therefore, our study is aimed at demonstrating the detailed molecular mechanism of UCA1 related to miRNA and transcription regulation, which will contribute in and enrich the UCA1 regulation mechanism in CRC metastasis.

In lncRNA regulatory function, the competitive endogenous RNA (ceRNA) network is critical in describing the lncRNA mechanism [5]. In CRC tumorigenesis and progression, the ceRNA network exhibits key roles, in which the lncRNA/miRNA/mRNA axis is a highlight. Previous studies clarified that UCA1 is involved in ceRNA regulations. In ovarian cancer, UCA1 targets miR-143 directly through 3′UTR binding, which causes the expression changes of FOSL2 [13]. Furthermore, UCA1 promotes prostate cancer progression through miR-143 targeting and forms a ceRNA relation with MYO6 [11]. Therefore, UCA1-mediated ceRNA networks are potential targets in preventing cancer progression. In this study, we validated the UCA1/miR-185/NOTCH3 regulatory axis in CRC.

Since lncRNA expression is also regulated by different transcription factors, here, we also performed analysis on the specific transcription factor of UCA1. In previous studies, several transcription factors have been identified to up- or downregulate UCA1. Ets-2, C/EBP*α*, TAZ/YAP/TEAD, and HIF-1*α* are transcription factors which upregulate UCA1. Transcription factors SATB1 and CAPER*α*/TBX3 downregulate UCA1 in cancers [14–19]. In this study, UCA1 was characterized as a direct target of HES5. The detailed regulations were also investigated.

## 2. Materials and Methods

### 2.1. Cell Culture

DLD-1, SW480, SW48, and HCT-116 cells were purchased from ATCC. HEK-293T cell was obtained from the cell bank of the Chinese Academy of Sciences. CRC cell lines were cultivated in McCoy's 5A medium (Gibco), and HEK-293T cell was cultivated in Dulbecco's modified Eagle medium (DMEM) (Gibco) containing 10% fetal bovine serum (FBS) (Invitrogen, Carlsbad, CA), 100 units/ml penicillin, and 0.1 mg/ml streptomycin in 20% O_2_ and 5% CO_2_ at 37°C.

#### 2.1.1. Vectors and Transfection

The HES5 CDS region was obtained through PCR and was cloned into pcDNA3.1 (+) vector (pcD-HES5). The inserted sequence was verified by Sanger sequencing. In transfection, cells were seeded into 6-well plates and cultivated overnight to over 75% confluence. Vectors, miRNAs, or siRNAs were transfected into cells by Lipofectamine RNAiMax (Thermo Fisher, USA) according to the provided instruction. In transfection, the final concentrations of vectors and RNAs were 1 *μ*g/ml and 25 nM.

#### 2.1.2. Quantitative Real-Time PCR (Q-PCR)

The High Pure RNA Isolation Kit (Roche, Switzerland) was used to extract total RNAs of treated cells. In reverse transcription, 500 ng total RNA was used to obtain cDNA. Q-PCR was conducted with SYBR® Premix Ex Taq™ (RR420A; TaKaRa) in the Roche LightCycler 480 system. The results were normalized to GAPDH and calculated by using the 2^−ΔΔ*Ct*^ value. The primers used in q-PCR analysis were listed in Table 1.

#### 2.1.3. Western Blot

Total protein was isolated from cells after different treatments by using RIPA lysis buffer. Protein concentrations of each group were quantified with BCA kit based on instructions. Total 30 *μ*g protein was loaded on 10% sodium dodecyl sulfate polyacrylamide gel (SDS-PAGE). The electrophoresis conditions have a constant voltage of 120 V for 120 min. Then, protein was transferred to polyvinylidene difluoride membrane from gel (300 mA, 90 min). Membranes were blocked by 5% milk in 1x TBST and then incubated with primary antibodies CDH1 (1 : 1000, Life, USA), VIM (1 : 1000, Santacruz, USA), NOTCH3 (1 : 1000, Abcam, USA), and *β*-actin (1 : 2000, Abcam, USA) overnight at 4°C. Next, secondary antibody in 5% milk was incubated with membranes at room temperature for 1 h. The LI-COR Odyssey Imaging System was used for blot imaging.

#### 2.1.4. Migration Assay

For migration ability measurement, wound healing assay was employed. Cells were seeded into 6-well plates and cultivated overnight. Cell surface wound was generated by scratching cells with a 10 *μ*l tip. Cell debris was removed by prewarmed PBS and then cultivated for 1 h. Wound images were taken after 1 h cultivation and recorded as 0 h timepoint. Cells were continuously cultivated for 24 h, and then images were taken, recorded as a terminal timepoint. The migration rate was calculated with the difference of distances between two timepoints and normalized to the control group. The relative migration ability was calculated by comparing the migration rate of each group to the control group.

#### 2.1.5. Transwell Assay

To detect cell invasion ability, transwell assay was conducted. After different treatments, cells were seeded into the upper chamber with 8 *μ*m membrane coated with Matrigel at the density of 5 × 10^4^ cells/chamber. Fresh media without FBS were filled in upper chambers while media with 10% FBS were filled in lower chambers. After 48 h cultivation, chambers were fixed with methanol for 15 min and then stained with 0.5% crystal violet for 15 min. Cells were carefully moved in lower chambers. Images were captured by a microscope. For quantification, stained transwells were washed with 800 *μ*l 0.1% Triton X-100 in 1x PBS and incubated for 30 min. Then, 100 *μ*l Triton X-100 in 1x PBS solution was collected from each sample to a 96-well plate and the absorbance OD value at 490 nm was measured with a microplate reader. The relative invasion ability was calculated by comparing the OD value of each group to the OD value of the control group.

#### 2.1.6. MTT Assay

For the cell proliferation rate, cells were seeded in 96-well plates in 100 *μ*l of medium at the density of 3000 cells/well. Three parallel wells were assigned for the indicated time points. MTT solution (5 mg/ml) was added to each well and continued to incubate for 4 hours. Medium and washed cells were removed with PBS twice. 150 *μ*l DMSO in each well was added, and then, the plate was shaken for 10 min. OD values were measured at 490 nm wavelength.

#### 2.1.7. Dual Luciferase Assay

The 3′-untranslated regions (3′ UTRs) of UCA1 and NOTCH3 containing wild-type (WT) or mutant (MUT) binding sites of miR-185 were cloned into the pGL3 control vector. For transcription factor validation, the mutation was performed and the promoter region was cloned into the pGL3 control vector. Luciferase assay was performed in HEK-293T cells. HEK-293T cells were seeded in 12-well plates at 3 × 10^4^ cells/well before transfecting with 100 ng of the indicated firefly luciferase reporter plasmid, 20 ng of Renilla reporter plasmid as a normalization control, and 25 nM of miR-185 mimics (QIAGEN) or a negative control oligonucleotide (QIAGEN) with the HiPerFect Transfection Reagent (QIAGEN) for 48 hours. The luciferase activity of each group transfected with different vectors was measured by a microplate reader and analyzed based on the protocol of the instruction of the Dual-Luciferase Reporter Assay Kit (Promega).

#### 2.1.8. ChIP-qChIP (Q-ChIP) Analysis

DLD-1 cells were used to confirm the transcription factor target. The crosslinking process and chromatin immunoprecipitation were performed according to the instruction provided in the QuikChIP(TM) Kit (Novus, USA). Q-PCR was conducted followed by ChIP. The HES5 antibody (ab19411, Abcam, USA) used for q-ChIP was diluted at 1 : 100. AchR was served as a negative control according to the previous study [20]. The primers used for q-ChIP were listed in Table 2.

#### 2.1.9. Statistics

SPSS 21.0 was used to calculate all the values (means ± standard error of the mean). Statistical analyses were analyzed with Student's *t*-test in a two-group comparison. The one-way analysis of variance (ANOVA) test was used to verify the significance among experimental groups. The statistical significance was *p* < 0.05.

## 3. Results

### 3.1. Long Noncoding RNA UCA1 Highly Expressed in Colorectal Cancer

**Figure 1:**
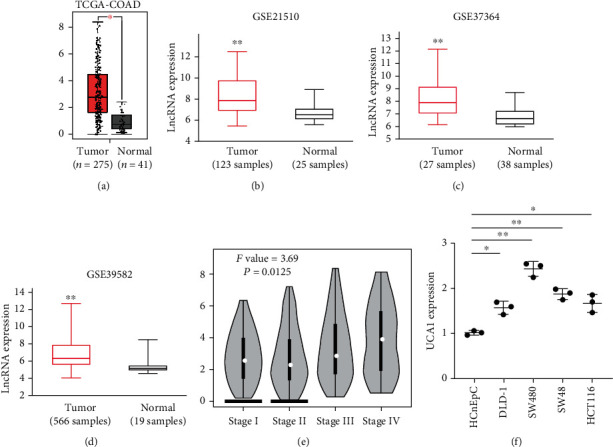
UCA1 was highly expressed in colorectal cancer. (a) The expression of UCA1 in CRC patients compared to that of normal tissue was obtained from TCGA-COAD database. (b–d) UCA1 expression in CRC tumor samples was confirmed in three independent cohorts from GSE21510, GSE37364, and GSE39582. (e) The expression of UCA1 in different CRC stages was confirmed based on TCGA-COAD database. Stage IV was compared to stage I. (f) Q-PCR analysis of UCA1 expression in four different CRC cell lines. The UCA1 expressions were compared to HCnEpC cells. ^∗^*p* < 0.05; ^∗∗^*p* < 0.01.

By analyzing the TCGA-COAD datasets, UCA1 was found to be highly expressed in samples with tumor burden compared to that in the normal group (Figure 1(a)). Furthermore, we analyzed three independent CRC cohorts (GSE21510, GSE37364, and GSE39582) obtained from GEO database. Results indicated that UCA1 was also highly expressed in CRC tumor tissues compared to normal samples (Figures 1(b)–1(d)). In addition, UCA1 expression was positively correlated to CRC advanced stages (Figure 1(e)). Next, we tested several CRC cell lines to validate UCA1 expression. Q-PCR analysis revealed that UCA1 expression was higher in CRC cell lines, especially in SW480 cell. Therefore, UCA1 is a tumorigenic lncRNA promoting CRC progression potentially.

### 3.2. Silencing UCA1 Inhibited Colorectal Cancer Cell Proliferation and Metastasis

**Figure 2:**
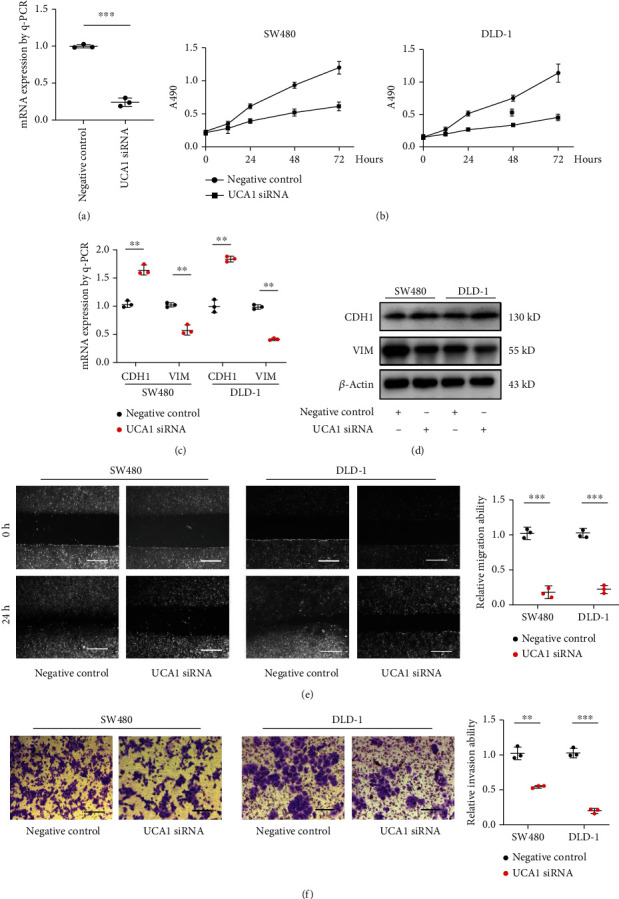
Silencing UCA1 reduced proliferation and metastasis of CRC cells. (a) Q-PCR analysis of UCA1 siRNA efficiency. (b) SW480 and DLD-1 cell proliferation rates were measured by MTT assay after being transfected with UCA1 siRNA in four time points. (c) The EMT maker expression level was confirmed by q-PCR analysis in SW480 and DLD-1 cells with UCA1 siRNA transfection. (d) The EMT maker protein level was confirmed by Western blot in SW480 and DLD-1 cells with UCA1 siRNA transfection. (e) The migration ability of SW480 and DLD-1 cells was tested by wound healing assay after being treated with UCA1 siRNA. Quantification was presented, scale bars: 100 *μ*m. (f) The invasion ability of SW480 and DLD-1 was confirmed by transwell assay after being transfected with UCA1 siRNA. Quantification was provided and three independent groups were included, scale bars: 20 *μ*m. ^∗∗^*p* < 0.01; ^∗∗∗^*p* < 0.001.

Since UCA1 had a significantly higher level in CRC cell lines, the function of UCA1 was studied. In SW480 cell, UCA1 was knocked down by the designed specific siRNA pool (Figure 2(a)). MTT assay revealed that UCA1 knocking down remarkably reduced proliferation rates of SW480 and DLD-1 cells (Figure 2(b)). Next, several epithelial-mesenchymal transition (EMT) markers were tested in SW480 and DLD-1 cells when UCA1 was silenced. Both q-PCR and Western blot results indicated that epithelial marker CDH1 (E-cadherin) was induced while mesenchymal marker VIM was inhibited by UCA1 siRNA (Figures 2(c) and 2(d)), suggesting that UCA1 promotes the EMT process in CRC cells. In addition, the migration ability of SW480 and DLD-1 cells was repressed by UCA1 siRNA compared to that of the negative control group (Figure 2(e)). The similar results were also observed in Boden chamber transwell assay, which illustrated that UCA1 silencing caused a less invasive ability in CRC cells (Figure 2(f)). The above results suggest that UCA1 promotes proliferation and metastasis of CRC cells.

### 3.3. miR-185 Directly Targeted UCA1

**Figure 3:**
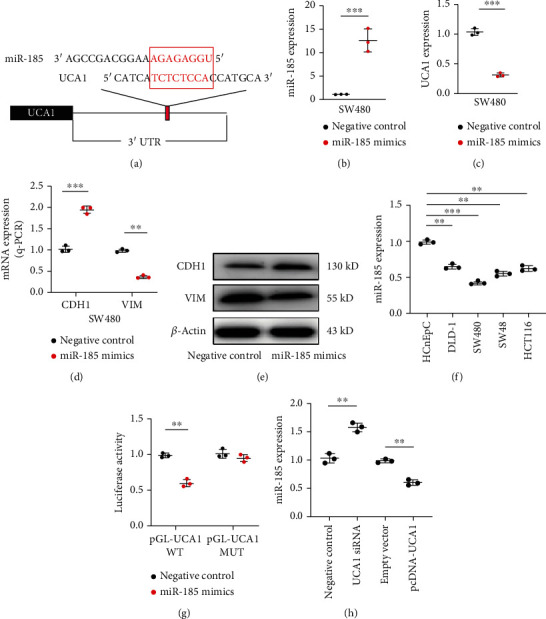
miR-185 directly targeted UCA1. (a) Target scheme of miR-185 and UCA1 3′UTR. The seed sequence of the target was highlighted with red. (b) Ectopic expression of miR-185 by miRNA mimics was confirmed by q-PCR analysis in SW480 cells. (c) UCA1 expression was inhibited by miR-185 confirmed by q-PCR analysis in SW480 cells. (d) The EMT maker expression level was confirmed by q-PCR analysis in SW480 with UCA1 miR-185 mimic transfection. (e) The EMT maker protein level was confirmed by Western blot in SW480 with UCA1 miR-185 mimic transfection. (f) Q-PCR analysis of miR-185 expression in four different CRC cell lines. The UCA1 expressions were compared to HCnEpC cells. (g) The target of miR-185 and UCA1 was confirmed by luciferase assay. (h) Q-PCR analysis of miR-185 expression after ectopically expressed UCA1 or silenced UCA1. ^∗∗^*p* < 0.01; ^∗∗∗^*p* < 0.001.

Next, the possible interactions between UCA1 and miRNAs were analyzed through bioinformatics tool ENCORI. The result showed that UCA1 3′UTR contained the miR-185 target seed sequence (Figure 3(a)). Then, miR-185 mimics were transfected into SW480 cells to achieve ectopic expression (Figure 3(b)). Ectopic expression of miR-185 significantly reduced the UCA1 level compared to that of the negative control group, validated by q-PCR analysis (Figure 3(c)). Meanwhile, q-PCR and Western blot analysis showed that CDH1 was induced but VIM was suppressed by ectopically expressed miR-185 (Figures 3(d) and 3(e)). Furthermore, the basal level of miR-185 was also observed to be downregulated in the indicated CRC cell lines (Figure 3(f)), suggesting that miR-185 is a tumor-suppressor miRNA. Dual luciferase assay validated that UCA1 was a direct target of miR-185 (Figure 3(g)). Interestingly, silencing UCA1 induced miR-185 expression, in which ectopic UCA1 expression repressed miR-185 expression (Figure 3(h)), which implied that UCA1 functions as a sponge of miR-185. In summary, UCA1 is a direct target of miR-185. UCA1 is a sponge of miR-185, which affects miR-185 expression.

### 3.4. UCA1 Modulated the miR-185/NOTCH3 Regulatory Axis

**Figure 4:**
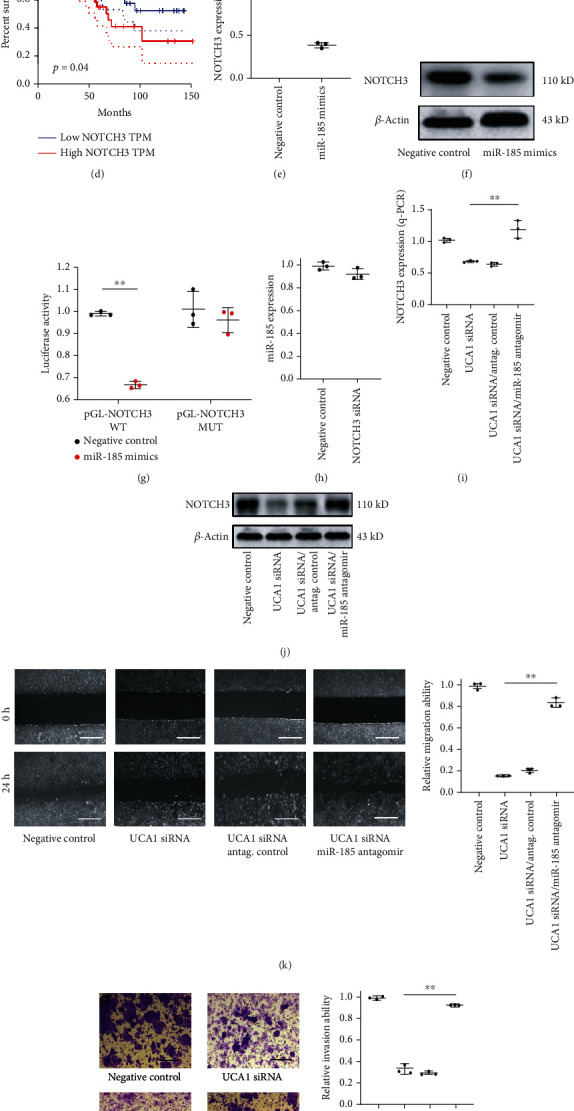
UCA1 modulated miR-185/NOTCH3 signaling. (a) Target scheme of miR-185 and NOTCH3 3′UTR. The seed sequence of the target was highlighted with red. (b) The expression of NOTCH3 in CRC patients compared to normal tissue was obtained from TCGA-COAD database. (c) The expression of NOTCH3 in different CRC stages was confirmed based on TCGA-COAD database. Stage IV was compared to stage I. (d) The relation between NOTCH3 expression and overall survival rate was analyzed based on the TCGA-COAD cohort. (e, f) NOTCH3 expression was inhibited by miR-185 confirmed by q-PCR analysis and Western blot in SW480 cells. (g) The target of miR-185 and NOTCH3 was confirmed by luciferase assay. (h) The effect of silencing NOTCH3 on miR-185 expression. (i, j) The effect of silencing UCA1 on NOTCH3 expression analyzed by q-PCR and Western blot. (k) SW480 cell migration ability tested by wound healing assay after the indicated different treatments. The quantification was also presented, scale bars: 100 *μ*m. (l) The SW480 cell invasion ability was tested by transwell assay after the indicated different treatments. The quantification was also presented, scale bars: 20 *μ*m. (m) The regulation model of UCA1, miR-185, and NOTCH3. ^∗^*p* < 0.05; ^∗∗^*p* < 0.01.

Since the ceRNA network is important for lncRNA function and regulation, here, we also investigated the potential gene which is possibly regulated by miR-185. Bioinformatics analysis revealed that NOTCH3 was a potential direct target of miR-185 (Figure 4(a)). In TCGA-COAD datasets, NOTCH3 was highly expressed in tumor tissues compared to those of the normal group (Figure 4(b)). Highly expressed NOTCH3 indicated advanced tumor stages and a poor overall survival rate (Figures 4(c) and 4(d)). Ectopic miR-185 expression repressed NOTCH3 mRNA and protein levels in SW480 cell (Figures 4(e) and 4(f)). The direct target between miR-185 and NOTCH3 was validated by dual luciferase assay since miR-185 inhibited the luciferase activity of the pGL3 reporter vector containing the NOTCH3 3′UTR wild-type sequence (Figure 4(g)). However, silencing NOTCH3 slightly changes the miR-185 expression level (Figure 4(h)), suggesting that NOTCH3 is under a one-way regulation by miR-185. Furthermore, silencing UCA1 in SW480 caused a lower NOTCH3 expression level compared to that of the negative control group, while miR-185 inhibition reversed this effect (Figures 4(i) and 4(j)), indicating that UCA1 regulates NOTCH3 via miR-185 mediation. Indeed, both migration ability and invasion ability of SW480 cells were reversed by miR-185 antagomir when UCA1 was silenced (Figures 4(k) and 4(l)), implying that UCA1 promotes SW480 cell metastasis through regulating the miR-185/NOTCH3 target. Collectively, UCA1 promotes CRC metastasis by upregulating NOTCH3, which is mediated by miR-185 (Figure 4(m)).

### 3.5. HES5 Was Validated as a Transcription Factor of UCA1

**Figure 5:**
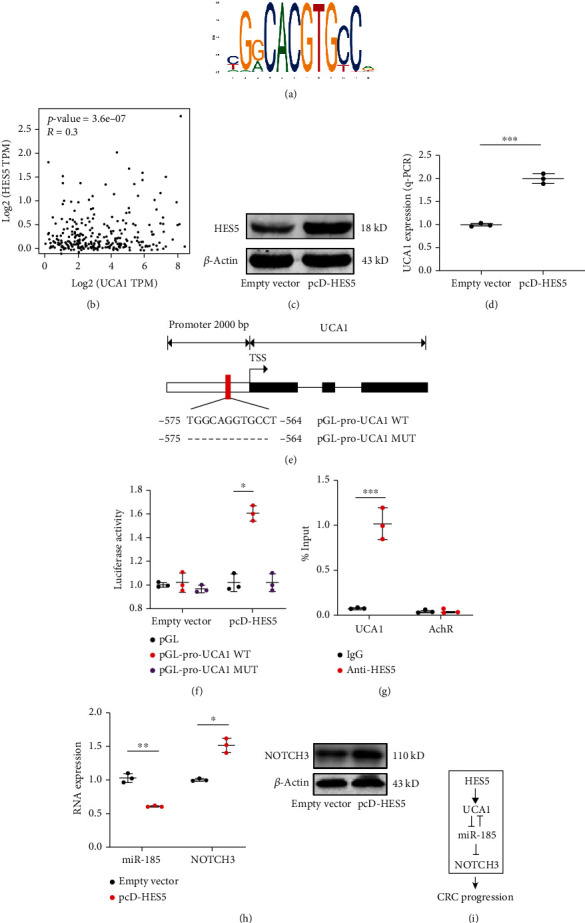
HES5 was validated as transcription factor of UCA1. (a) JASPAR predicted the putative HES5 binding motif in the human UCA1 promoter. (b) The expression correlation between UCA1 and HES5 expression in CRC tissue obtained from TCGA-COAD database. (c) Ectopic expression of HES5 by pcD-HES5 overexpression plasmid. (d) UCA1 expression was induced by HES5 overexpression confirmed by q-PCR. (e) and (f) The putative binding site of HES5 on UCA1 located in UCA1 promoter region indicated with red. The target was confirmed by luciferase assay. (g) UCA1and HES5 target was validated by q-ChIP analysis. AchR was served as a negative control. (h) miR-185 and NOTCH3 expression was confirmed by q-PCR analysis after ectopically expressed HES5. NOTCH3 expression was also tested by Western blot. (i) Regulation model of HES5, UCA1, miR-185 and NOTCH3 in CRC progression. ∗p < 0.05, ∗∗p < 0.01, ∗∗∗p < 0.001.

To further clarify UCA1 regulation in CRC, the potential transcription factor was investigated. Here, the 2000 bp promoter region of UCA1 was obtained from the UCSC genome browser and scanned in JASPAR to discover potential transcription factors. As shown in Figure 5(a), HES5 was predicted as a putative transcription factor of UCA1 by binding with the indicated typical motif. From the analysis in TCGA-COAD datasets, HES5 expression was positively correlated to UCA1 expression in CRC (Figure 5(b)). In HEK-293T cells, overexpression of HES5 induced the UCA1 level (Figures 5(c) and 5(d)). To confirm the target between HES5 and UCA1, we performed dual-luciferase assay on the pGL vector containing the UCA1 promoter region mutated following the indicated strategy (Figure 5(e)). The mutant binding motif of UCA1 exhibited a lower luciferase activity compared to that of the wild-type group, suggesting that HES5 regulates UCA1 expression through activating transcription (Figure 5(f)). In addition, q-ChIP analysis demonstrated that the UCA1 promoter region was enriched by HES5 precipitation, validating the direct target between UCA1 and HES5 (Figure 5(g)). Furthermore, overexpression HES5 repressed miR-185 and induced NOTCH3 expression in HEK-293T cells (Figure 5(h)). In summary, UCA1 is a direct target of HES5. NOTCH3 is regulated by HES5 through the HES5/UCA1/mIR-185 axis.

## 4. Discussion

Colorectal cancer is the third most commonly diagnosed cancer around the world, which caused over 800000 deaths in 2018 [2]. One of the challenges in CRC clinical treatment is metastasis which results in poor prognosis and high recurrence rate. Recent studies indicate that lncRNA exerts regulatory functions in CRC progression. In previous studies, UCA1 has been identified to serve as a tumorigenic lncRNA in different cancer types such as bladder cancer, breast cancer, lung cancer, melanoma, and colorectal cancer [6–11]. Furthermore, UCA1 is considered as a biomarker indicating a poor prognosis, metastasis, and drug resistance [21]. Here, UCA1 was confirmed to be highly expressed in colorectal cancer. Moreover, the UCA1 expression level was positively correlated to tumor stages. Silencing UCA1 showed an inhibitory effect on cell proliferation and metastasis. Therefore, UCA1 is a oncogenic lncRNA promoting CRC progression.

In this study, we also performed analysis on the miRNA-UCA1 target. Previous studies have shown that UCA1 is targeted by several miRNAs. In hepatocellular carcinoma, miR-216b targets UCA1 and represses cancer progression by inactivating the ERK signaling pathway [22]. UCA1 promotes bladder cancer cell migration and invasion through miR-145 targeting [23]. In other cancer types, UCA1 were also identified as targets of miR-1, miR-16, miR-18a, and miR-204 [24–27]. Here, we validated that UCA1 is a direct target of miR-185. Indeed, miR-185 plays a tumor-suppressive role in cancer [28]. Therefore, the miR-185/UCA1 target is important for CRC progression. Interestingly, UCA1 overexpression repressed miR-185 expression, indicating that UCA1 also serves as a miRNA sponge.

In lncRNA regulation, the competitive endogenous RNA (ceRNA) network is critical in describing the lncRNA function mechanisms [5]. In current research, NOTCH3, the member of the NOTCH signaling pathway, was validated as a direct target of miR-185. In CRC, NOTCH3 has been identified to drive tumor progression. Increased NOTCH3 was observed in CRC and associated with accelerated tumor growth [29]. Here, NOTCH3 shared the same miR-185 target with UCA1. In addition, NOTCH3 was indued by UCA1 through miR-185 mediation. Therefore, CRC is promoted by the UCA1/miR-185/NOTCH3 regulatory axis.

Besides the ceRNA regulatory network, we also performed analysis on the potential transcription factor of UCA1. Previous studies have identified that UCA1 is regulated by several transcription factors such as Ets-2, C/EBP*α*, TAZ/YAP/TEAD, HIF-1*α*, SATB1, and CAPER*α*/TBX3 [14–19]. In this study, HES5 was characterized as a transcription factor of UCA1. In the UCA1 promoter region, the binding motif of TGGCAGGTGCCT was confirmed to be critical for HES5 transcription ability. Furthermore, NOTCH3 was also induced by HES5 via the HES5/UCA1/miR-185/NOTCH3 regulatory axis. Therefore, UCA1 plays a central role in mediating HES5, miR-185, and NOTCH3 function in CRC progression.

In conclusion, UCA1 promotes CRC metastasis and proliferation. UCA1 is a direct target of HES5. Both UCA1 and NOTCH3 are direct targets of miR-185, through which NOTCH3 is induced by UCA1, summarized in Figure 5(i).

## Figures and Tables

**Table 1:** Primers used in q-PCR analysis.

Primer name	Primer sequence 5′ to 3′
UCA1 Fwd.	ACGCTAACTGGCACCTTGTT
UCA1 rev.	CTCCGGACTGCTTCAAGTGT
CDH1 Fwd.	CGAGAGCTACACGTTCACGG
CDH1 rev.	GGGTGTCGAGGGAAAAATAGG
VIM Fwd.	AGTCCACTGAGTACCGGAGAC
VIM rev.	CATTTCACGCATCTGGCGTTC
NOTCH3 Fwd.	CGTGGCTTCTTTCTACTGTGC
NOTCH3 rev.	CGTTCACCGGATTTGTGTCAC

**Table 2:** Primers used in q-ChIP.

Primer name	Primer sequence 5′-3′
UCA1 qChIP Fwd.	GGAGGCCAGCCGGTGGAT
UCA1 qChIP rev.	GAGACAGAGTCTTGATCTGTTGCCC
AchR Fwd.	CCTTCATTGGGATCACCACG
AchR rev.	AGGAGATGAGTACCAGCAGGTTG

## Data Availability

The datasets generated during and/or analyzed during the current study are available from the corresponding author on reasonable request.
